# Inter-patient image registration algorithms to disentangle regional dose bioeffects

**DOI:** 10.1038/s41598-018-23327-0

**Published:** 2018-03-20

**Authors:** Serena Monti, Roberto Pacelli, Laura Cella, Giuseppe Palma

**Affiliations:** 1 IRCCS SDN, Napoli, Italy; 2 Department of Advanced Biomedical Sciences, “Federico II” University School of Medicine, Napoli, Italy; 3 Institute of Biostructures and Bioimaging, National Research Council, Napoli, Italy

## Abstract

Radiation therapy (RT) technological advances call for a comprehensive reconsideration of the definition of dose features leading to radiation induced morbidity (RIM). In this context, the voxel-based approach (VBA) to dose distribution analysis in RT offers a radically new philosophy to evaluate local dose response patterns, as an alternative to dose-volume-histograms for identifying dose sensitive regions of normal tissue. The VBA relies on mapping patient dose distributions into a single reference case anatomy which serves as anchor for local dosimetric evaluations. The inter-patient elastic image registrations (EIRs) of the planning CTs provide the deformation fields necessary for the actual warp of dose distributions. In this study we assessed the impact of EIR on the VBA results in thoracic patients by identifying two state-of-the-art EIR algorithms (Demons and B-Spline). Our analysis demonstrated that both the EIR algorithms may be successfully used to highlight subregions with dose differences associated with RIM that substantially overlap. Furthermore, the inclusion for the first time of covariates within a dosimetric statistical model that faces the multiple comparison problem expands the potential of VBA, thus paving the way to a reliable voxel-based analysis of RIM in datasets with strong correlation of the outcome with non-dosimetric variables.

## Introduction

Elastic Image Registration (EIR) has recently gained momentum in the field of Radiation Therapy (RT). In particular, intra-patient EIR plays an integral role in modern Treatment Planning (TP) strategies, such as the Image Guided RT, which exploits the acquisition of several patient imaging datasets during the course of RT. In this context, intra-patient EIR has paved the way to real-time plan re-optimization^1^ thanks to its capability to automatically track the contoured structures over time or to perform a dose warping. Intra-patient EIR was also interestingly used to study the RT-induced texture changes in serial computed tomography (CT) scans^2^ or for 4D CT dose accumulation at different respiratory phases^3^.

Besides the relatively common application of intra-patient EIR to TP, the inter-patients version of EIR has been more recently exploited in population analysis to correlate local dose and radiation-induced morbidity (RIM). RIM has historically been estimated by condensing the 3D dose distribution into a monodimensional dose-volume histogram (DVH) that disregards spatial information of the dose. Increasing evidences, however, suggest that a voxel-based approach (VBA) allows to identify unprecedented correlations between RIM and local dose release. In particular, VBAs were applied to identify local dose effects for rectal^4^, gastrointestinal^5^ and urinary toxicity^6,7^ in prostate cancer patients. In addition, VBA was demonstrated suitable for unveiling potential spatial signatures of radiation sensitivity in inhomogeneous organs – such as the lungs^8^ – or in composite regions – such as the head and neck district^9^. Of note, so far none of the previous VBAs took into account non-dosimetric variables, such as patients’ or treatment-related characteristics.

A key issue of a fully 3D VBA is represented by the EIR to a common anatomical reference (the so called *spatial normalization*) of the analyzed cohort of patients. The schemes for EIR most commonly used in medical imaging or RT applications are the Demons and B-spline algorithms^10–14^.

In the context of RIM analyses, the Demons approach has been shown to guarantee excellent performance in inter-patient EIR, with a robust match of anatomical structures both from a pure geometrical point of view and in terms of dose-organ overlap^4,8,9^. On the other hand, the B-spline parameterization approach was exploited in the spatial normalization of a cohort of lung cancer patients included in a survival analysis^15^.

To the best of our knowledge, none of the previous studies explored the influence of the EIR algorithm on highlighting the regional dose effects on RIM.

Given this background, the main focus of the present study was a comparative analysis of the regional dose effects on RIM when the Demons and B-spline algorithms are used within the VBA framework. The method was applied to a cohort of patients followed up for late lung toxicity after thoracic irradiation. We tested the null hypothesis of group differences (patients with RIM versus patients without RIM) on both physical dose and biologically effective dose (BED)^16^. Moreover, non-dosimetric variables were tested for significant correlation with RIM, and for the first time the VBA was designed to take into account their potential influence.

## Methods and Materials

### Patients’ dataset

For the present analysis, we have considered a cohort of *N* = 98 patients treated for Hodgkin lymphoma (HL) with post-chemotherapy supradiaphragmatic involved-field 3D-conformal RT. A median treatment total dose of 30.6 Gy (range: [20.8, 45.0] Gy) in daily fractions of 1.5–1.8 Gy was prescribed. All patients were followed up for late pulmonary toxicity according to the Radiation Therapy Oncology Group scoring system^17^. At a median time to event of 13 months (range: [9, 83] months), 18 patients displayed radiation-induced CT radiological density changes (the RIM actually considered in the present analysis). All participants gave written informed consent and the patient data were analyzed anonymously. This retrospective study was approved by the local Ethics Committee (Comitato Etico per le Attività Biomediche, Università Federico II, Napoli, n. 222–10). All experimental protocols and procedures were performed in accordance with the guidelines of the Università Federico II, Napoli. Details on patients’ and treatment characteristics (Supplementary Table) were previously reported^18^.

The contours of lung tissue and heart were reviewed on planning CTs following RTOG 1106^17^ and heart atlas contouring guidelines^19,20^. The CT matrix size was 512 × 512 in plane with a slice thickness of 5 mm.

### Voxel-based approach

Individual DICOM RT plans (CT scans, doses and contoured organs) were converted into a Matlab (MathWorks, Natick, MA)-readable format using the CERR (Computational Environment for Radiotherapy Research) software^21^. BED maps were voxelwise extracted from dose maps for late toxicity effects (α/β = 3 Gy) according to^22^. All processing steps described below were handled using in house software developed in Matlab.

The VBA consists of two main processes^8,9^:

1. Spatial normalization of the patients’ cohort, whose core part consists in the EIR of the planning CTs to a common anatomical reference and the consistent warp of the associated doses and BEDs;
2. Statistical analysis of regional dose differences between patients with and without RIM.

The patient with the median lung volume was chosen as the common anatomical reference (*i*.*e*. the common coordinate system – hereafter CCS) for the spatial normalization of the cohort. Before running the actual EIR of all other patients on the CCS, in order to enhance both the robustness and the efficiency of a potentially cumbersome registration task, CT scans were pre-processed as follows. For each patient, a binary mask of the region of interest was computed as the union and dilation (by a spherical structuring element of radius 30 mm) of heart and lung TP structures. The field-of-view was cropped accordingly and a coarse alignment of the structures of interest was obtained by an affine registration based on the mask boundary. CT images were also masked, in order to hide some inter-individual or gender-related anatomical differences of limited interest to our study, and to have the EIR algorithms work more effectively on tissue contrast inside the chest.

Then, the actual EIR was performed by open source implementations of two different algorithms: the B-spline Elastix registration^23^ with Adaptive Stochastic Gradient Descent (ASCD) optimization algorithm, and the log diffeomorphic extension of the Demons registration^24^; both methods were implemented with a multi-resolution strategy and adopted the mutual information as loss function. First, the patients’ CTs were registered to the CCS, taking advantage of the anatomical details provided by the structural imaging; then, the obtained deformation fields were used to warp the dose and BED maps into the CCS as well. It should be emphasized that, since spatial normalization only aims at anchoring the next statistical analysis to a known and patient-independent anatomy, no Jacobian intensity modulation was applied to the deformed doses and BEDs.

The median Hausdorff distances between the union *M* of heart and lung structures of the CCS and the corresponding spatially normalized unions of the *N*-1 patients were calculated for each EIR algorithm. These defined the full width at half maximum of the two spherical Gaussian kernels used to smooth the spatially normalized dose and BED maps^25^ for the following regional statistical analysis.

Next, the statistical analysis constituting the second main process of the VBA was performed according to a test for general linear models (GLMs) counteracting the multiple comparison problem inherent in the task. In particular, a non-parametric permutation test^26^ based on the Threshold Free Cluster Enhancement^25^ of a maximum-*T* statistics allows for an excellent compromise between the sensitivity of the analysis and the control over imagewise Type I errors.

In order to adjust the GLM for possible non-dosimetric covariates, a univariate analysis of their differences among patients with and without RIM was first performed by Pearson’s *χ*^2^- or Fisher’s exact test (categorical variables) or Mann-Whitney *U*-test (continuous variables). A multivariate analysis with backward stepwise selection (based on the Wald statistic) was then performed including any variable having a univariate test *p*-value ≤ 0.25^27^.

Afterwards, an extension of the former GLM was designed by adding a new regressor for each variable left in the regression model.

The voxel-based statistical analysis was performed by means of the “randomise” tool available in the FMRIB Software Library v5.0 (https://fsl.fmrib.ox.ac.uk/fsl/fslwiki/FSL)^28^. The output of each voxel-based analysis was a *p*-map of dose or BED differences between the groups of patients with and without RIM, possibly adjusted for potential covariates.

### Data analysis

The accuracy of the two different spatial normalizations was evaluated by computing the Dice Index (DI)^29^, the modified Hausdorff distance (MHD)^30^, the dose-organ overlap (DOO) index^4^ and the Root Mean Squared Error (RMSE). Wilcoxon signed rank test was used to compare each score achieved by the two different EIRs.

To evaluate the impact of EIR algorithm on the results of VBA, a Minkowski distance between two *p*-maps, *A* and *B*, was computed as

1 $${d}_{a}(A,B)={(\frac{1}{\lambda (M)}{\int }_{M}{|A-B|}^{a}d\lambda )}^{1/a}$$

(*λ*(*M*) being the measure of the union of lungs and heart), and two further concordance metrics of the *p*-maps were *ad hoc* devised. The first one aims at evaluating the matching of the significance regions of two *p*-maps (*e*.*g*. the *p*-maps obtained by the VBA test without adjustment for covariate on the BED maps spatially normalized with the two EIRs) at a given confidence level. Denoting by *S*_*P*_ the sublevel set of a *p*-map for a given *P* value, the DI over *p* (DI*p*) between two *p*-maps *A* and *B* was defined for each min(min(*A*), min(*B*)) < *P* ≤ 1 as:

2 $${\rm{DI}}p[A,B](P)={\rm{DI}}({S}_{P}[A],{S}_{P}[B])$$

The second metrics provide a similar hint, except that the match is evaluated for an equal volume of the significant regions, instead of equal confidence level. Strictly speaking, if *f*_*X*_(*P*) is the relative volume of *S*_*P*_[*X*], the DI over volume (DIV) was defined for each 0 < *P* ≤ 1 as:

3 $${\rm{DIV}}[A,B](V)={\rm{DI}}({S}_{{f}_{A}^{-1}(V)}[A],{S}_{{f}_{B}^{-1}(V)}[B])$$

### Data availability

The data analyzed in the present study are available at http://www.ibb.cnr.it/?command=viewcms&id=216.

## Results

The paired comparison analysis of the DI, MHD, DOO and RMSE values (Table 1) computed on the whole cohort for Demons and B-spline showed that significantly better scores were obtained by the B-spline registration algorithm.

**Table 1:** Registration scores pre- and post-elastic image registration by B-spline and Demons algorithms.

Structure		DI			MHD (mm)			DOO			RMSE (Hounsfield Units)		
		pre	B-spline	Demons	pre	B-spline	Demons	pre	B-spline	Demons	Pre	B-spline	Demons
**Lungs**	Median	0.77	0.96	0.94	2.55	0.15	0.33	0.59	0.90	0.87	357	96	114
	Range	[0.55,0.88]	[0.93,0.97]	[0.85,0.95]	[0.73,10.72]	[0.07,3.62]	[0.21,2.05]	[0.32,0.78]	[0.83,0.93]	[0.74,0.92]	[223,485]	[73,193]	[93,194]
	*p*-value^§^		<10^−16^			<10^−15^			<10^−16^			<10^−12^	
**Heart**	Median	0.71	0.89	0.82	4.05	0.88	3.46	0.57	0.79	0.70	209	35	39
	Range	[0.22,0.87]	[0.69,0.93]	[0.70,0.89]	[0.97,24.31]	[0.30,4.48]	[1.00,7.60]	[0.06,0.85]	[0.26,0.88]	[0.23,0.88]	[38,534]	[29,71]	[33,78]
	*p*-value^§^		<10^−15^			<10^−15^			<10^−8^			<10^−14^	

DI = Dice Index, MHD = Modified Hausdorff Distance, DOO = Dose-Organ Overlap, RMSE = Root Mean Squared Error.

^§^*p* values refer to the comparisons between B-spline and Demons algorithms.

Age was the only non-dosimetric variable selected by multivariate regression analysis with backward elimination (*p*=0.012). Median age in patients with RIM was 32 yr (range: [22, 69] yr) vs 27 yr (range: [13, 58] yr) in patients without RIM. Age was therefore included within the GLMs adjusted for possible covariates.

**Figure 1:**
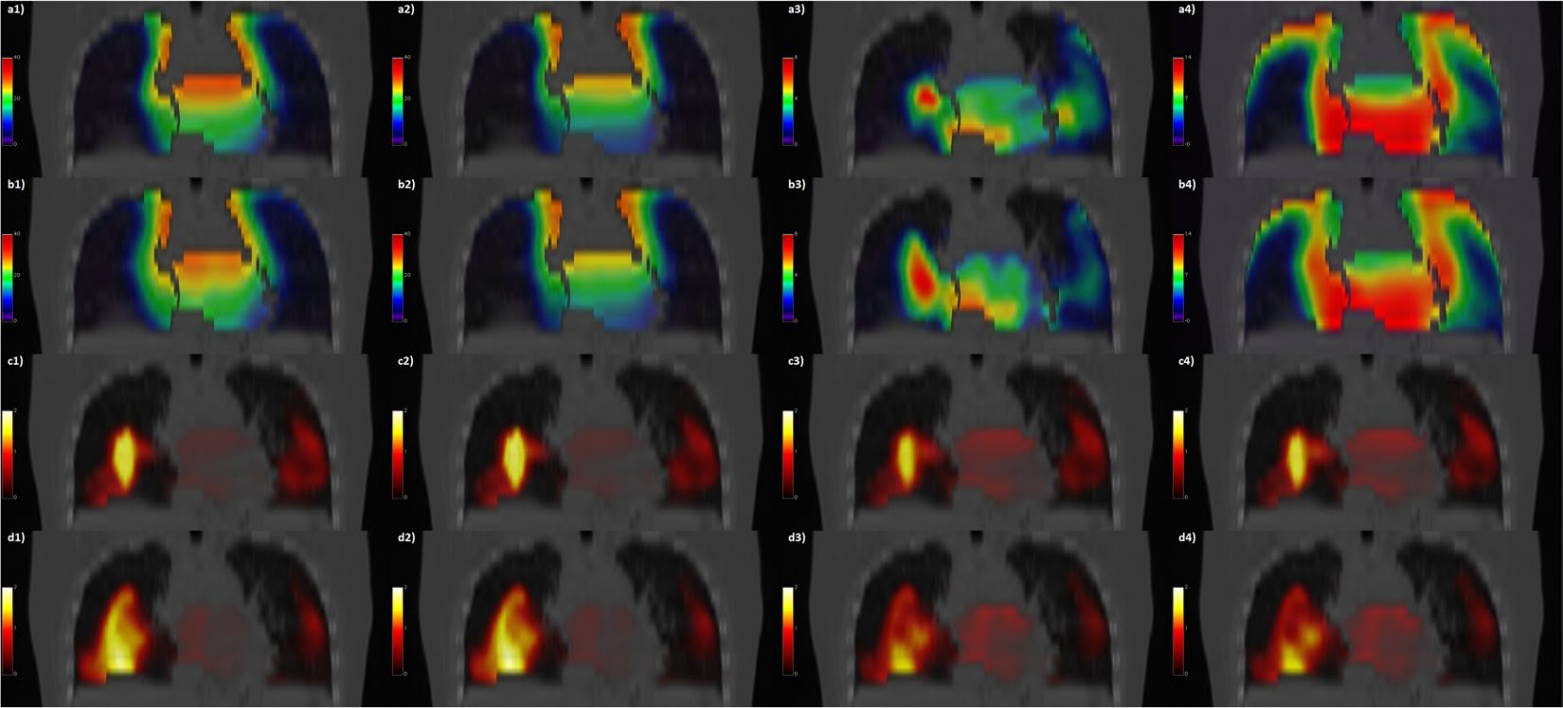
Coronal views of the thorax CT fused with dose-related maps (rows *a* and *b*) and with significance p-maps (rows *c* and *d*). Columns in rows *a* (B-spline) and *b* (Demons) show: 1) mean dose maps (Gy) for patients with RIM; 2) mean dose maps (Gy) for patients without RIM; 3) dose difference between 1) and 2); 4) dose standard deviation map within the cohort. Please note that color maps in columns 3) and 4) have different scales of 1) and 2). Columns in rows *c* (B-spline) and *d* (Demons) show the maps of -Log(*p*) as derived from tests for: 1) GLM on dose; 2) GLM on BED; 3) GLM on dose and age; 4) GLM on BED and age.

One significance map was obtained by the VBA for each of the eight triads (Fig. 1) given by the choice between B-spline or Demons (C1), dose or BED (C2), and adjustment for age or not (C3). All the *p*-maps^4^ highlight clusters of significant dosimetric differences between the two groups of patients, *i*.*e*. all *S*_0.05_[*X*_*i*_] were non-empty (Table 2).

**Table 2:** Minimum *p* value and volume of significant cluster at *p* = 0.05 level for the analyzed configurations.

GLM		Dose		BED	
		B-spline	Demons	B-spline	Demons
No age	*p*_min_	0.017	0.011	0.016	0.009
	*f*(0.05)	44.7 cc	118 cc	49.9 cc	93.7 cc
Age	*p*_min_	0.018	0.022	0.017	0.017
	*f*(0.05)	30.8 cc	46.6 cc	37.0 cc	51.1 cc

*Abbreviations*: GLM = General Linear Model, BED = Biologically Effective Dose.

**Figure 2:**
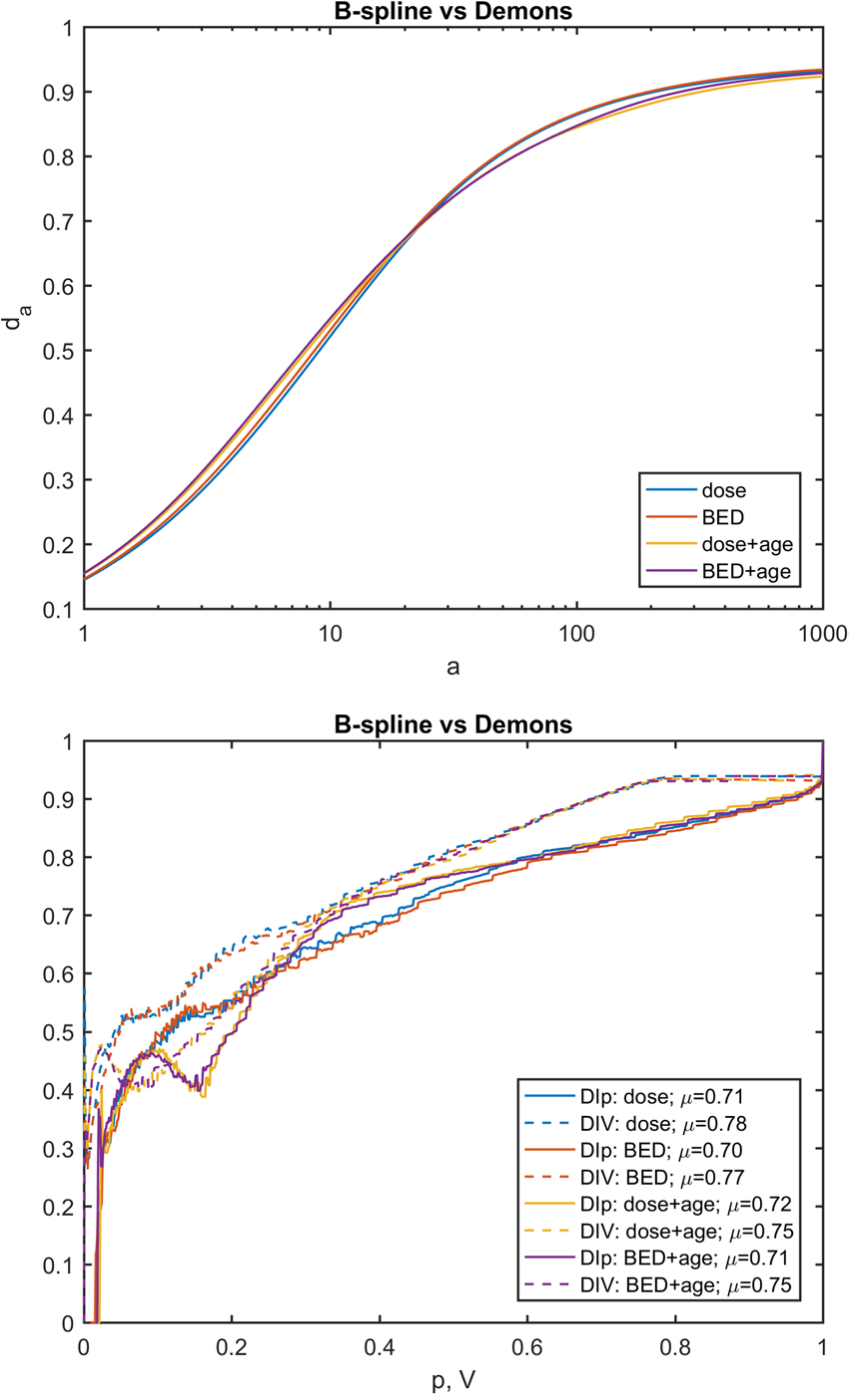
Plots of concordance metrics *d*_*a*_, DI*p* and DIV (computed for each of the four pairs of choices C2 and C3) for comparison of p-maps derived from B-spline and Demons registration algorithms. In the legend, *μ* is the mean of the function over its domain.

**Figure 3:**
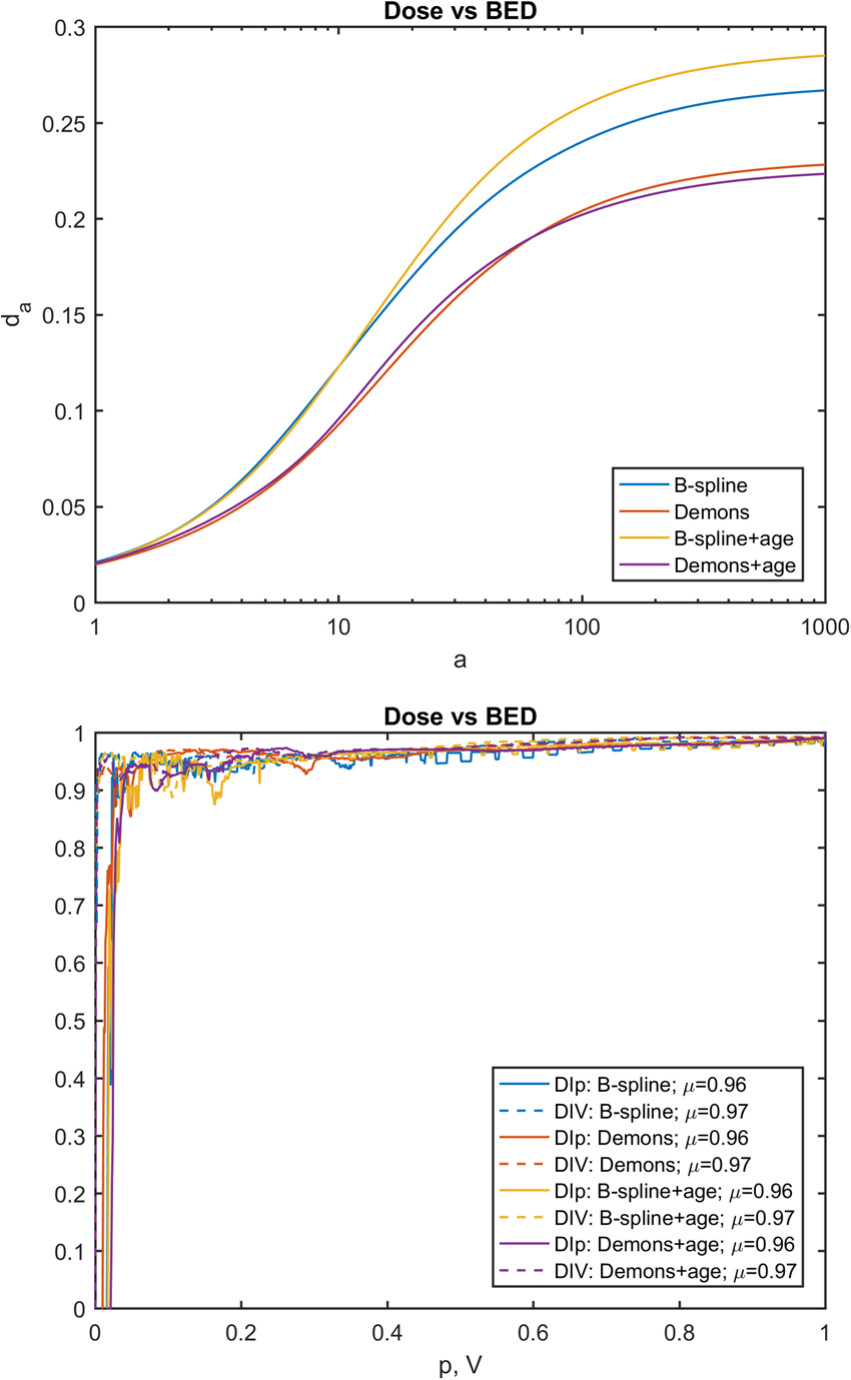
Plots of concordance metrics *d*_*a*_, DI*p* and DIV (computed for each of the four pairs of choices C1 and C3) for comparison of p-maps derived for dose and BED. In the legend, *μ* is the mean of the function over its domain.

**Figure 4:**
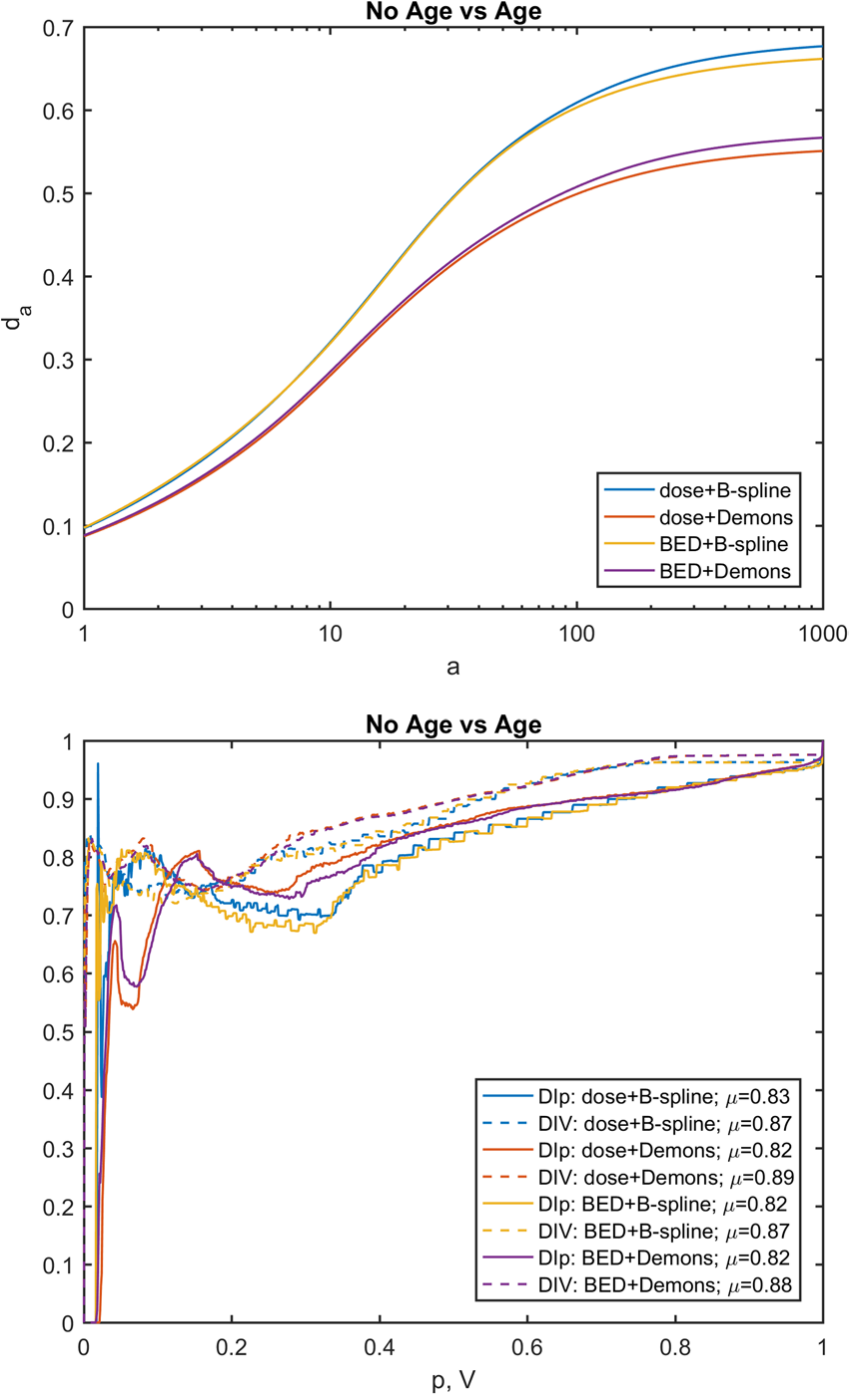
Plots of concordance metrics *d*_*a*_, DI*p* and DIV (computed for each of the four pairs of choices C1 and C2) for comparison of p-maps derived from GLM adjusted or not for age. In the legend, *μ* is the mean of the function over its domain.

A visual inspection suggests that all the maps highlight clusters of high statistical significance in overlapping regions. The highest source of variability among the results is associated with the choice of the EIR algorithm, with a smaller influence exerted by the inclusion of age within the GLM, and an almost negligible effect of switching between dose and BED. This is confirmed by the quantitative analysis of the *p*-map concordance (Figs 2–4): when switching from C1 to C3 and from C3 to C2, *d*_*a*_ curves tend to decrease, while DI*p* and DIV show a consistent positive trend in the integral means of the functions.

## Discussion

A quite large amount of EIR tools are nowadays available for intra- or inter-patient warping of structural datasets^10^. Many of them can be substantially classified as belonging to one of two transformation models: the Demons and the B-spline. Among these, we selected two highly effective algorithms for which an open-source implementation is available: the log diffeomorphic extension of the Demons registration^24^ and the B-spline Elastix registration^23^. Both approaches are known to have pros and cons^31^. Diffeomorphic Demons ensure the invertibility of the deformation fields and allow following complex warps thanks to the high number of degrees of freedom; conversely, the same high number of degrees of freedom leads to a potential sensitivity to noise. On the other hand, B-spline transforms, in spite of their flexibility, need some special care in order to ensure that non-invertible deformations are unlikely to occur^32^: proper regularization terms can be added to the cost function to ensure smoothness and avoid singularities in the deformation field, or appropriate optimization algorithms (such as the ASCD adopted in this work) can be chosen to avoid local minima associated to undesirable deformation^33^.

This study deals with a problem, namely the VBA, which requires reliability at an inter-patient level of the EIR process. This makes it intrinsically more challenging than tasks resting on an intra-patient registration. Indeed, the need to compare doses released in the same anatomical location to different patients demands the capability to overcome the high variability of examined structures potentially related to individual characteristics (gender, height, weight, etc.).

The VBA, performed by both EIR algorithms, was able to highlight a local dose-RIM relationship in the lungs, suggesting that the irradiation of peripheral parenchymal region in the middle and caudal lung is correlated with RIM (Fig. 1). In particular, as previously discussed in Palma *et al*.^8^, a higher dose was delivered in the low-dose parenchymal regions, in agreement with some recent DVH analyses showing that the lung volume exceeding 5 Gy is consistently more predictive for RIM than other dosimetric variables^18,34^.

A large body of literature addressed the issue of quantifying the accuracy of the different registration algorithms under the action of a variety of deformation fields on a ground truth and according to several fidelity metrics^10^. Differently, our aim was to evaluate the overall impact of the EIR algorithms on the final output of the VBA pipeline. We believe that a non-trivial test bench can be the thorax region, due to the poor structural CT information content in relatively large scales of lung parenchyma. Moreover, the high inter-individual anatomical variations of bronchi and of related pulmonary vessels^35^ hinder the exploit of the vessel CT contrast as a reliable landmark for the deformation process.

As a first step, we evaluated the EIR performance in heart and lungs by means of DI and MHD scores, both accounting for pure geometric match; in addition, DOO score was computed in order to weight the DI by the involved doses. It should be emphasized that these scores only give hints on boundary mismatch between structures, but are not able to measure the consistency of the deformation model well within the considered organs. In this respect, the RMSE is designed to take into account the anatomical match inside the organs. Both B-spline and Demons achieved results absolutely satisfactory in comparison with the pre-registration scores. Moreover, B-spline significantly outperformed Demons in all the considered metrics.

When we considered the entire VBA pipeline, the overlap of significant regions of dose-related differences – confirmed by both a visual inspection (Fig. 1) and a quantitative analysis of the *p*-maps (Figs 2–4) – at first justifies our confidence that results obtained by a VBA are quite stable against the choice of the EIR algorithm. Nonetheless, the adoption of the Demons or the B-spline scheme raises differences in the details of the significance pattern of dosimetric differences associated with the RIM development. This offers food for thought regarding the anatomical detail than can be achieved by the hitherto proposed VBA schemes. Namely, it seems confirmed that VBA allows for confidently going beyond the detail of an organ-based DVH approach; however, there is a limit to the level of substructures that can be identified.

This argument equally applies when we aim at considering the additional effects of BED or the inclusion of non-dosimetric covariates. Indeed, the comparison among the variability induced in the *p*-maps by C1, C2 and C3 configurations highlights that effects associated with C2 (dose or BED) and C3 (adjustment for age or not) fall below the influence of the EIR algorithm choice. That being said, we recognize that the extent of observed effects of C2 and C3 may be highly dependent on the tested outcome, the considered patients’ cohort and irradiation schemes. For instance, in this case, the low ratio between dose fraction in healthy tissues and α/β is likely to be responsible for the low influence we observed on *p*-maps when correcting the physical dose for biological effects.

The adoption of a VBA can have a twofold goal: the identification of the patterns of high radiosensitivity within organs and the definition of avoidance regions for sophisticated TP strategies^9^. This provides the rationale behind the definition of DI*p* and DIV metrics to compare two *p*-maps: on the one hand, DI*p* measures the concordance between clusters at the same significance level; DIV, instead, given that a tradeoff between the risk of RIM and tumor dose coverage has to be established, measures the match between the volumes at higher RIM risk for a given extension of the avoidance region.

In conclusion, in the present work we performed a comprehensive analysis of the impact of EIR on VBA, demonstrating that both the state-of-the-art EIR algorithms may be successfully used to highlight subregions with dose differences associated with RIM that substantially overlap, with the exception of small scale discrepancies. Furthermore, we expanded the potential of VBA by showing that covariates can be included within a statistical model facing the multiple comparison problem, thus paving the way to a reliable voxel-based analysis of RIM in datasets with strong correlation of the outcome with non-dosimetric variables.

We believe that it should be relevant to the radiation oncology community whether the recent findings of several pioneering studies^4,8,9,15^ are stable against the choice of the tool used in a key image processing step. In particular, as the regional dose patterns in RIM or survival analysis are receiving more and more attention, it is clinically crucial to lay robust methodological foundations for the whole voxel-based approach.

## Electronic supplementary material

Supplementary Table
