# Supplementary material for: Inter-patient image registration algorithms to disentangle regional dose bioeffects
Source: Sci Rep. 2018 Mar 20;8:4915. doi: 10.1038/s41598-018-23327-0 (PMC5861107; doi:10.1038/s41598-018-23327-0)
Supplement: Supplementary file 1 — Supplementary Table [file 41598_2018_23327_MOESM1_ESM.docx]

**Inter-patient image registration algorithms to disentangle regional dose bioeffects**

Serena Monti^1^, Roberto Pacelli^2^, Laura Cella^3*^, Giuseppe Palma^3^

^1^ IRCCS SDN, Napoli, Italy

^2^ Department of Advanced Biomedical Sciences, “Federico II” University School of Medicine, Napoli, Italy

^3^ Institute of Biostructures and Bioimaging, National Research Council, Napoli, Italy

**Corresponding author**: Laura Cella, Institute of Biostructures and Bioimaging, National Research Council (CNR), Via T. De Amicis, 95, 80145 – Napoli, Italy. Tel: +39-081-2203427; E-mail: laura.cella@cnr.it

**Supplementary Table**. Results of univariate analysis on clinical variables

|  | | |  | | **Univariate Analysis** |
| --- | --- | --- | --- | --- | --- |
| Characteristics | | | |  | *P*-value |
| **Continous variables** | | | | Median (range) |  |
|  | Age (yr) | | | 28 (13-69) | .019 |
|  | Total Lung Volume (cm^3^) | | | 2645 (1363-5729) | .521 |
|  | Left Lung Volume (cm^3^) | | | 1236 (664-2793) | .237 |
|  | Right Lung Volume (cm^3^) | | | 1446 (732-3048) | .811 |
|  | Heart Volume (cm^3^) | | | 524 (336-1459) | .862 |
| **Categorical variables** | | | | N (%) |  |
|  | Gender | | |  |  |
|  | | Female | | 59 (60.2) |  |
|  | | Male | | 39 (39.8) | 1.0 |
|  | Histology | | |  |  |
|  | | Nodular sclerosis | | 78 (79.6) |  |
|  | | Mixed cellularity | | 19 (19.4) |  |
|  | | Lymphocyte-rich-classical | | 1 (1.0) | 0.88 |
|  | Stage | | |  |  |
|  | | I-II | | 79 (80.6) |  |
|  | | III-IV | | 19 (19.4) | .520 |
|  | Chemotherapy regimen | | |  |  |
|  | | ABVD | | 30 (30.6) |  |
|  | | VEBEP | | 66 (567.3) |  |
|  | | BEACOPP | | 2 (2.0) | .319 |
|  | Risk factors | | |  |  |
|  | | None | | 72 (73.5) |  |
|  | | Yes | | 26 (26.5) | .609 |

*Abbreviations*: ABVD= doxorubicin, bleomycin, vinblastine and dacarbazine; VEBEP= vinblastine, etoposide, bleomycin, epidoxorubicin.

*Note*: Risk factors: smoking habit, diabetes, hypertension, cardiac comorbidities. There can be multiple risk factors simultaneously. Yes: patients with at least 1 risk factor. None: patients without risk factors.
